# Secure and Lightweight Cloud-Assisted Video Reporting Protocol over 5G-Enabled Vehicular Networks

**DOI:** 10.3390/s17102191

**Published:** 2017-09-23

**Authors:** Lewis Nkenyereye, Joonho Kwon, Yoon-Ho Choi

**Affiliations:** School of Computer Science and Engineering, Pusan National University, Busan 46241, Korea; nkenyele@pusan.ac.kr (L.N.); jhkwon@pusan.ac.kr (J.K.)

**Keywords:** 5G cellular network, cloud assisted vehicular networks, security, video reporting

## Abstract

In the vehicular networks, the real-time video reporting service is used to send the recorded videos in the vehicle to the cloud. However, when facilitating the real-time video reporting service in the vehicular networks, the usage of the fourth generation (4G) long term evolution (LTE) was proved to suffer from latency while the IEEE 802.11p standard does not offer sufficient scalability for a such congested environment. To overcome those drawbacks, the fifth-generation (5G)-enabled vehicular network is considered as a promising technology for empowering the real-time video reporting service. In this paper, we note that security and privacy related issues should also be carefully addressed to boost the early adoption of 5G-enabled vehicular networks. There exist a few research works for secure video reporting service in 5G-enabled vehicular networks. However, their usage is limited because of public key certificates and expensive pairing operations. Thus, we propose a secure and lightweight protocol for cloud-assisted video reporting service in 5G-enabled vehicular networks. Compared to the conventional public key certificates, the proposed protocol achieves entities’ authorization through anonymous credential. Also, by using lightweight security primitives instead of expensive bilinear pairing operations, the proposed protocol minimizes the computational overhead. From the evaluation results, we show that the proposed protocol takes the smaller computation and communication time for the cryptographic primitives than that of the well-known Eiza-Ni-Shi protocol.

## 1. Introduction

Due to the merits of the fifth-generation (5G) cellular networks such as higher mobility support, massive connectivity and reduced latency [1], the academia and industry have shown interest in the 5G technology as a shifting paradigm which overcomes the limitations of the fourth generation (4G) technology. For example, leading companies in IT such as Cisco has predicted that the 5G cellular networks could meet the global mobile data traffic projections for the next five years [2]. That is, it is expected that the 5G cellular networks through the *massive connectivity* property will enable the connection of millions of devices including vehicles.

Especially, 5G cellular networks were embraced as the ultimate framework which would help the implementation of vehicular related technologies [3,4,5]. For example, a driverless vehicle was tested over 5G cellular networks by *Uber* in the beginning of the year 2017 [6]. Let us note that vehicular network is still in its architectural stage regardless of considerable amount of research works available in the literature [7,8,9,10]. Also, security and privacy threats, lack of scalability and latency due to the high mobility of the vehicles are considered as the main reasons that delay the real deployment of vehicular networks. In practice, the researchers have proved that even IEEE 802.11p lacks of mobility support [7] and the Long Term Evolution (LTE) network does not support the effective latency for the vehicular networks [11,12,13]. Thus, the predicted performance of 5G cellular network in terms of latency, mobility and so on has been considered as a promising archetype for the practical implementation of the intelligent transportation system (ITS)-related services through the cloud assisted vehicular network.

Before the full deployment of the 5G cellular network in the year 2020 [14], security and privacy related issues should be carefully addressed to boost its adoption [15,16]. Furthermore, the ITS services based on the innovative 5G cellular network will require strong security because the data packets are relayed in the safety-critical vehicular environment [17]. That is, the design of secure protocols for the 5G-enabled ITS-related services is required.

To send the videos recorded by the vehicle’s cameras into the cloud server, we propose a secure and lightweight cloud-assisted video reporting protocol for 5G-enabled vehicular networks. Recently, Eiza et al. [18] and Yoo [19] have proposed the secure cloud-assisted video reporting protocols in the 5G-enabled networks. By including handover and certificate revocation algorithms, Yoo enhanced the Eiza-Ni-Shi protocol that was designed by using public key certificates. In this paper, we focus on resolving the following drawbacks of the Eiza-Ni-Shi protocol:

- The Eiza-Ni-Shi protocol relies on convectional public key certificates that should be renewed in the vehicle periodically, e.g., every year. However, such periodic renewal is proved to be burdensome over the vehicular networks [20,21,22].
- The Eiza-Ni-Shi protocol is built on expensive pairing operations. Thus, the overall efficiency of the Eiza-Ni-Shi protocol can be decreased despite the merits of 5G cellular networks.
- The Eiza-Ni-Shi protocol is designed by using an attribute-based encryption. When attribute-based encryption is used to achieve access control, the video sender should know the public key of the receiver. This preliminary makes the Eiza-Ni-Shi protocol to be only applicable in limited services.

Also, we note that the cloud-assisted video reporting protocol should be designed to fulfill security requirements such as privacy, authorization and fine-grained access control over the vehicles. For example, the sender’s personal data should not be revealed to unauthorized entities or even the reporting vehicles should not be traced by any malicious users. Even under the huge computation capabilities of 5G-enabled vehicular network, the cloud entities should not waste much time and computation capabilities before they discard bogus, unauthenticated and unauthorized videos. In addition, for the proposed protocol to attain the *non-repudiation* property, the conditional traceability of all the vehicles should be achieved.

To fulfill the above-mentioned goals of 5G-enabled cloud-assisted video reporting protocol and to overcome the drawbacks of the Eiza-Ni-Shi protocol, we propose a new secure and lightweight video reporting protocol for 5G-enabled vehicular networks. We summarize the contributions of this work as follows:

- We define an application model for a secure and lightweight cloud-assisted video reporting protocol over 5G-Enabled vehicular networks. The model highlights the security objectives that the protocol should satisfy within the 5G-Enabled vehicular networks architecture.
- We develop a secure and lightweight cloud-assisted video reporting protocol for 5G-enabled vehicular networks. Without using the conventional public key certificates, the proposed protocol supports entities’ *authorization* through anonymous credential. Since the reported videos are broadcasted by the fixed entities, the designated vehicles can recover the reported videos without making any time-consuming communication. Also, by using lightweight security primitives, the proposed protocol minimizes the computation overhead and meets the performance requirement for the real-time ITS-based services in 5G-Enabled vehicular networks.
- We evaluate the performance of the proposed protocol in terms of security objectives, computation cost and communication overhead.

The rest of this paper consists of as follows. After we describe the related work in Section 2, cryptographic primitives for constructing the proposed protocol are overviewed in Section 3. After describing the overall operation of the proposed protocol in Section 4, we show the detailed operations in Section 5. In Section 6, we show the performance analysis results of the proposed protocol. Finally, we conclude this paper in Section 7.

## 2. Related Work

In this section, we overview the evolution of vehicle communication architectures for supporting the video reporting service in 5G-enabled vehicular networks.

### 2.1. VANETs and 5G-Enabled Cloud-Assisted VANETs

As an extension of mobile ad-hoc networks (MANETs) [23], the main entities in vehicular ad hoc networks (VANETs) include the vehicles, the fixed infrastructures along the roads, called road side units (RSU), and an over-viewer third party, called Trusted Authority (TA), in charge of registration, certification and revocation of all the entities within the VANETs architecture. Commonly, VANETs architecture is classified into two main communication means namely vehicle-to-vehicle (V2V) and vehicle to infrastructure (V2I) [24]. The computational cost for the value-added applications in VANETs requires huge computation capabilities, which led to the mixture of VANETs and cloud computing, called *VANETs using Cloud* [25]. *VANETs using Cloud* is defined as vehicular networks equipped with smart devices which communicate with the cloud in the same way as our mobile phones connect to different servers located in the cloud. *VANETs using Cloud* was introduced in [26] by Olariu et al. for the first time. Olariu et al. suggested an autonomous vehicular cloud (AVC) architecture as a special case of *VANETs using Cloud*. In [27], Hussain et al. presented additional services by combining cloud computing and VANETs: Computing as a Service (CompaaS), Storage as a Service (STaaS), Network as a Service (NaaS), Cooperation as a Serivce (CaaS), Entertainment as a Service (ENaaS), Information as a Service (INaaS) and Traffic-Information as a Service (TIaaS). Hussain et al. pointed out the feasibility of *VANETs using Cloud* compared to convectional VANETs. Also, the feasibility of *VANETs using Cloud* was approved by several researchers [28,29,30,31,32]. *Vehicular Cloud* refers to the full utilization of vehicle devices as computers to form mobile servers. In this architecture, one could use the vehicle’s OBU to make his/her personal cloud. As a combination of *Vehicular cloud* and *VANETs using Cloud*, *Hybrid vehicle cloud* was proposed. The proposed protocol is built on *VANETs using Cloud* framework, i.e., cloud-assisted vehicular networks. In the following sections, vehicular networks and cloud-assisted vehicular networks are used interchangeably.

### 2.2. Security and Video Reporting in 5G Enabled Vehicular Network

Security and privacy related topics in 5G cellular networks have mostly being dedicated to security threats of each of the distinct technology (SDN or NFV) [33,34]. Among relevant works on security concerns over 5G cellular network, Mantas et al. [35] surveyed probable threats and attacks against the core modules of 5G cellular networks. The authors also confirmed the four conventional attractive targets in the 4G cellular network: user equipment (UE); access network; the mobile operator’s core network; and external Internet Protocol networks. Yang et al. [36] suggested that much effort should be paid on the physical layer of the 5G cellular network. The malicious users are likely to take advantage of the deficiencies of the wireless communication medium such as the poor signal reception quality. Some notable solutions proposed by the authors on the physical layer include artificial noise, confidential and antenna correlation. Alam et al. [37] proposed a framework that analyzes the security requirements of the three scenarios of device to device (D2D) communications in LTE Advanced (LTE-A) networks. The first one includes the network-covered D2D communication without user applications, in which all the nodes in the proximity are under an LTE-A network coverage and the user applications do not need D2D communications. The following scenario is the network-covered D2D communication, where all the devices including the users’ devices are covered by an LTE-A network. The last scenario is the network-absent D2D communication. For all these three types of D2D scenarios, conventional security attacks such as eavesdropping, impersonation attack and the corresponding countermeasure solutions were introduced [37].

For vehicular environment, several researches have addressed potential security and privacy issues in VANETs [38,39,40,41]. ITS based services such as navigation services received much attention for the last decade [22,42]. Other protocols in the literature addressed multiple services in VANETs [43,44,45] for the VANETs integrated with cloud computing. However, all the afore-mentioned protocols are not based on HetNet architecture such as 5G-enabled vehicular network, where our protocol is built upon. Recently, researchers in [18,19] introduced secure and privacy aware cloud-assisted video reporting service in 5G-Enabled vehicular network. However, as noted in Section 1, their protocols have some limitations. This, the design of a new secure and lightweight cloud-assisted video reporting protocol over 5G-enabled vehicular networks is required.

## 3. Preliminary

We overview the preliminary security properties such as attribute-based encryption (ABE) and certificateless signature scheme.

### 3.1. Attribute-Based Encryption Scheme

The ABE scheme in [46] is designed for elliptic curve cryptography and is made of the following sub-protocols: Setup, Encryption, Key-Generation, and Decryption algorithms.

#### 3.1.1. ABE.Setup

For the universe of attributes U={1,2,…,n}; let $G_{1}$ be an additive group with a prime order *q* and $P\in G_{1}$, where $G_{1}$ is made of points on an elliptic curve and *P* is a generator of $G_{1}$. ABE.Setup() sub-protocol works as follows:

- On input of a random $s\in Z_{q}^{*}$ as the attribute master secret key, output the corresponding public key PK=s·P.
- For each i∈U, choose an attribute secret $l_{i}\in Z_{q}^{*}$ to generate the attribute public key $P_{i}=l_{i}\cdot P$.
- Set $ABEmk=\{s,l_{1},\ldots ,l_{\left|U\right|}\}$ and $ABE.params=\{PK,P_{1},\ldots ,P_{\left|U\right|}\}$.
- Returns $\langle ABEmk,ABE.params\rangle$.

#### 3.1.2. ABE.ENC

For a message *m*, ω as attribute set, and ABE.params, ABE.ENC(*m*, ω, ABE.params) returns the ciphertext CM as follows:

- On input of $k\in Z_{q}^{*}$, then output the key K=k·PK.
- Compute $C=Enc_{K}\left(m\right)$.
- For i∈ω, compute $W_{i}=k\cdot P_{i}$, respectively.
- Output the ciphertext $CM=\langle \omega ,C,\{W_{i}\;|\;i\in \omega \}\rangle$.

#### 3.1.3. ABE.KGN

ABE.KGN(ABEmk, Γ) algorithm outputs the shared secret for the decryption keys under the attribute set ω, which consists of a master secret ABEmk and the access tree Γ.

- Based on access tree Γ, allocate index to every node other than root.
- A polynomial $q_{node}\left(x\right)$ over $Z_{q}^{*}$ is set in top-down manner for each node where each polynomial is of degree $d_{node}-1$ and $d_{node}$ is considered as the threshold value of the node.
  − Set $q_{root}\left(0\right)=s$ for the root node.
  − Set $q_{node}\left(0\right)=q_{parent}\left(index\left(node\right)\right)$ for every node with a leaf, where index(node) represents the node’s index value.
- Suppose Γ contains *n* leaves, for every leaf node $leaf_{f}$ (1≤f≤n), a secret share for the decryption key is generated as $D_{leaf_{f}}=q_{leaf_{f}}\left(0\right)\cdot t_{i}^{-1}$ where *i* represents the attribute linked to $leaf_{f}$ and $l_{i}$ a random number for *i* taken in ABE.Setup.
- Output $D=\{D_{leaf_{f}}\;|\;leaf_{f}\in \Gamma \}$.

#### 3.1.4. ABE.DEC

ABE.DEC(CM, *D*, ABE.params) performs the decryption of the cipher text CM, as long as the attributes set ω fulfills the access tree Γ, by using NodeKey(CM, *D*, node) for every node within the access tree recursively. In this ABE scheme [46], secret sharing based on Lagrange interpolation is borrowed to recover the decryption key.

- For every leaf node linked to an attribute *i*, NodeKey(CM, *D*, $leaf_{f}$) is computed as follows. Note that we represent NodeKey(CM, *D*, $leaf_{f}$) into $N_{1}$ in the next paragraph for convenience.
  1. In case the associated attribute *i* to $leaf_{f}$ is not comprised in ω, then NodeKey(CM, *D*, $leaf_{l}$) = ⊥.
  2. else,
    $$\begin{array}{ccc} N_{1} & = & D_{leaf_{f}}\cdot W_{i}\\ & = & q_{leaf_{f}}\left(0\right)\cdot t_{i}^{-1}\cdot k\cdot P_{i}\\ & = & q_{leaf_{f}}\left(0\right)\cdot t_{i}^{-1}\cdot k\cdot l_{i}\cdot P\\ & = & q_{leaf_{f}}\left(0\right)\cdot k\cdot P \end{array}$$
- To proceed with a non-leaf node *u*, the algorithm calls NodeKey(CM, *D*, *z*) for all children *z* which are attached to the node *u*.
  1. Suppose that $\omega_{u}$ is an arbitrary $d_{u}$ set of children nodes satisfying NodeKey(CM, *D*, *z*) ≠ ⊥. In case no such set is existent, NodeKey(CM, *D*, *u*) output ⊥. We use *N* to denote NodeKey(CM, *D*, *u*) in the next paragraph for paper formatting.
  2. Else, let $\Delta_{index\left(z\right),\omega_{u}^{\prime }}=\prod_{j\in \omega_{u}^{\prime },j\neq index\left(z\right)}\frac{x-j}{i-j}$ represent the Lagrange coefficient with $\omega_{u}^{\prime }=\left\{index\left(z\right)\;|\;z\in \omega_{u}\right\}$,
    $$\begin{array}{ccc} N & = & \sum_{z\in \omega_{u}}\Delta_{index\left(z\right),\omega_{u}^{\prime }}\left(0\right)\cdot \mathrm{NodeKey}\left(CM,D,z\right)\\ & = & \sum_{z\in \omega_{u}}\Delta_{index\left(z\right),\omega_{u}^{\prime }}\left(0\right)\cdot q_{z}\left(0\right)\cdot k\cdot P\\ & = & \sum_{z\in \omega_{u}}\Delta_{index\left(z\right),\omega_{u}^{\prime }}\left(0\right)\cdot q_{parent}\left(index\left(z\right)\right)\cdot k\cdot P\\ & = & \sum_{z\in \omega_{u}}\Delta_{index\left(z\right),\omega_{u}^{\prime }}\left(0\right)\cdot q_{u}\left(index\left(z\right)\right)\cdot k\cdot P\\ & = & q_{u}\left(0\right)\cdot k\cdot P \end{array}$$
- Compute the decryption key *K* = NodeKey(CM, *D*, root) = $q_{root}\left(0\right)\cdot k\cdot P=s\cdot k\cdot P$.
- Output the decrypted message $m=Dec_{K}\left(C\right)$.

### 3.2. Certificateless Signature Scheme

Certificateless signature scheme (CertS) [47] consists of the following procedures.

- CertS.Setup() computes a master key along with a public system parameters as follows:
  − Let *G* be an additive group with a prime order *q* and P∈G be a generator based on an elliptic curve.
  − Choose $s\in Z_{q}^{*}$ as master secret key and generates the master public key $P_{pub}=s\cdot P$.
  − Let $H_{1}:{\left\{0,1\right\}}^{*}\times G\to Z_{q}^{*}$ and $H_{2}:{\left\{0,1\right\}}^{*}\times G\to Z_{q}^{*}$ be two cryptographic hash functions.
  − Output the public parameters $CertS.params=\{G,q,P,P_{pub},H_{1},H_{2}\}$.
  − Output $\langle s,CertS.params\rangle$
- CertS.Secret(id) returns a secret value for every identity id as follows:
  − Choose $x_{id}\in Z_{q}^{*}$ as a secret value, then compute $P_{id}=x_{id}\cdot P$.
  − Return $Sec_{1}=\langle x_{id},P_{id}\rangle$
- CertS.PartialK(*s*, id, $P_{id}$) computes a partial private/public key for the given id as follows:
  − Select a random $r_{id}\in Z_{q}^{*}$ and generate $R_{id}=r_{id}\cdot P$.
  − Compute $s_{id}=r_{id}+s\cdot H_{1}\left(id,R_{id},P_{id}\right)$ (mod *q*).
  − Output $Sec_{2}=\langle s_{id},R_{id}\rangle$ as the corresponding partial private key.
- CertS.SKey($Sec_{1}$, $Sec_{2}$) sets $sk_{id}=\langle x_{id},s_{id}\rangle$ and $pk_{id}=\langle P_{id},R_{id}\rangle$ representing the private key and public key for id, respectively.
- CertS.Sign(*m*, $sk_{id}$) computes the signature for a given message *m* as follows:
  − Select a random $l\in Z_{q}^{*}$ such that gcd(l+h, *q*) = 1, where $h=H_{2}\left(m,R,P_{id},R_{id}\right)$ and R=l·P.
  − Generates $r={\left(l+h\right)}^{-1}\left(x_{id}+s_{id}\right)$ (mod *q*).
  − Output the signature $\sigma =\langle r,R\rangle$.
- CertS.Verify(*m*, id, $pk_{id}$, σ) provides the signature verification σ for the message *m* for the identity id as follows:
  − Generate $h_{1}=H_{1}\left(id,R_{id},P_{id}\right)$ and $h_{2}=H_{2}\left(m,R,id,P_{id},R_{id}\right)$.
  − Check whether $r\cdot \left(R+h_{2}\cdot P\right)\overset{?}{=}P_{id}+R_{id}+\left(h_{1}\;\cdot P_{pub}\right)$.

## 4. Overview of Proposed Protocol

After overviewing the system architecture and describing the security requirements, we explain the overall operation of the proposed protocol.

### 4.1. System Architecture

As a major difference from the convectional cloud assisted vehicular network architecture, the 5G-Enabled vehicular network is deployed on the following communication mediums:

- Heterogeneous Networks: This network is originated from the ultimate desire to achieve high data rate and network capacity for the 5G-enabled network. Thus, two solutions may help to attain the aforementioned capacities by making the size of cells smaller and embracing the mmWave spectrum. Making the size of the cell much smaller would increase the spectral efficiency [48]. On the other side, the mmWave communications will offer high data rates because it operates in the range of 30–300 GHz and 1–10 mm for the spectrum and wavelength respectively. As mentioned in [38], the mmWave technology still suffers from considerable propagation loss that generates tremendous line of sight (LOS) connections.
- D2D Communications: D2D communication enables devices to communicate with each other within the licensed cellular bandwidth without involving the BS. In the 5G-Enabled vehicular networks, the vehicles can communicate through D2D communication or by direct link under the mmWare technology.

**Figure 1:**
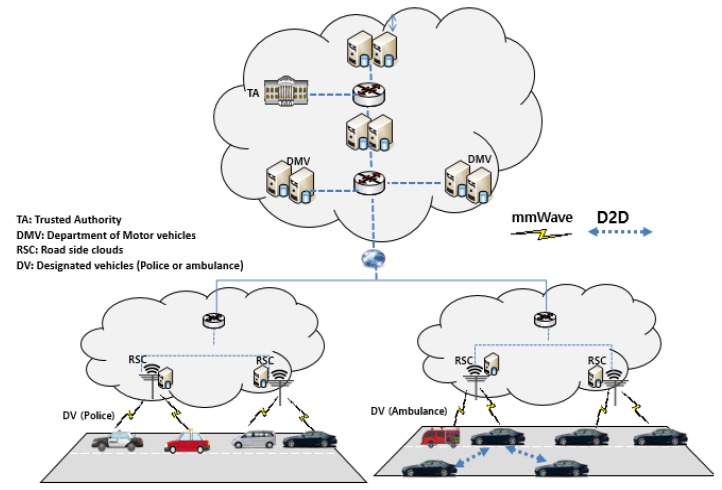
System Architecture.

We describe the communication entities in the proposed protocol: TA, DV, DMV, RSC and vehicles that communicate through the on board unit (OBU) as shown in Figure 1.

- Trusted Authority (TA): It is in charge of the registration of all entities (DMV, DV, RSC and vehicles) inside our system and issues cryptographic materials during the system initialization.
- Department of Motor Vehicles (DMV): All the vehicles are assumed to register with the DMV periodically. Beside the conventional techniques for vehicle’s identification including the Electronic License Plate (ELP) or the Electronic Chassis Number (ECN), each vehicle is registered with a 5G identifier (5GID) with the same functionalities as the subscriber identification module (SIM) chip.
- Road Side cloud (RSC): RSCs are servers located along the roads and accessible by the vehicles. RSCs stores the videos files (VFs) sent by the vehicles. In that case, the designated vehicles (DV) such as police or ambulance can download the files through the RSCs using mmWare communications. Due to the advancements of technology, we assume that RSCs are connected to an electricity power generator with enough computational capability.
- Designated Vehicles: The designated vehicles can be public or private vehicles registered by the government through the DMV that offers public services.
- Vehicles: Vehicles are equipped with OBUs which allow them to communicate with RSCs in order to send the recorded video files.

### 4.2. Security Objectives

The proposed protocol is designed to satisfy the following security requirements.

- Authentication and Authorization: Any vehicle has to be authenticated before it can send (report) a video recorded by its camera.
- Identity privacy preservation: The real identity of every vehicle should be protected from being known by other vehicles, RSC, DVs and DMV.
- Fine-grained access control: ABE should guarantee a fine-grained access control by which a designated vehicle should strictly be capable to recover a video fitting to its possessed access structure.
- Non-repudiation: A given vehicle should not deny its participation in video reporting.
- Traceability: TA should be capable of disclosing the real identity of all the entities in the system.

### 4.3. Overall Operation

The overall operation of the proposed protocol consists of system setup, periodic credential generation, on-duty token generation and accident Reporting procedures.

- System setup: TA sets up its master secret key and its corresponding public key. Each vehicle provides its real identity and TA generates the corresponding pseudo identity from which a partial private key is computed. DMVs and RSCs also provide their real identities and TA computes their partial private keys. Each vehicle registers with the TA through the DMV, the designated vehicles such as the police or ambulance also register with the TA through the DMV.
- Periodic credential generation: Periodically, a vehicle request for credential in order report the recorded videos. DMV generates the periodic credential to vehicles along with the set of attributes corresponding to the type of request. We assume that based on some criteria such as accident record or reckless driving, DMV can decide to give different set of attributes to participating vehicles.
- On-duty token generation: The designated vehicles also received on-duty tokens. These tokens will be used by the designated to retrieve reported videos from the RSCs.
- Accident Reporting: Periodically, a vehicle registers for road reporting services. During the registration, the vehicle specifies the types of services to be reported such as accident or abnormal scene (we assume that the camera of a vehicle is not limited to report road’s accidents only). An incentive technique based on point accumulation could be considered in order to motivate the vehicle users to participate in video reporting. Whenever an accident or abnormal scene occurs the camera records the scene and upload the files to the RSC using mmWare technology or D2D communication. The designated vehicles would later on acquire the report from the RSCs as long as they possess enough access structure to recover the secret.

## 5. Details of Proposed Protocol

**Figure 2:**
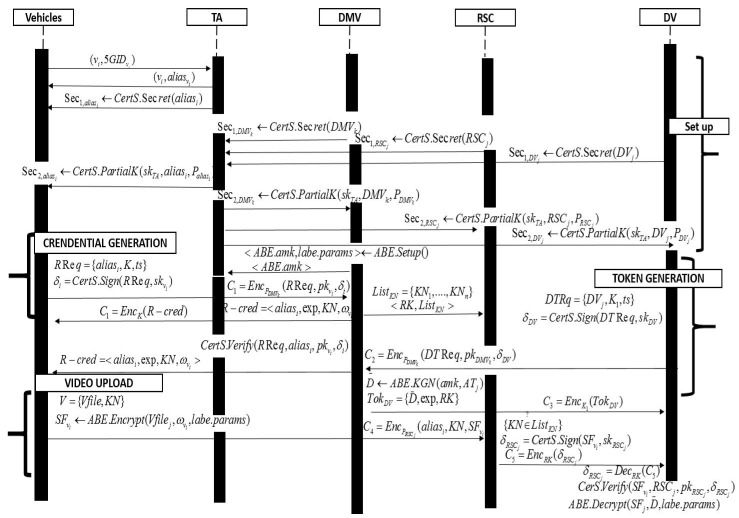
Protocol description.

In this section, we describe the secure and lightweight cloud assisted video reporting protocol over 5G-enabled vehicular networks in details. In Table 1, we summarize the notations used for the proposed protocol and the overall operations of the protocol are depicted in Figure 2.

### 5.1. System Setup

In setup phase, TA generates global system parameters and all the entities register to the TA as follows:

1. TA selects an elliptic curve group $G_{1}$ of order *q* with $P\in G_{1}$ as a generator.
2. In order to get the master secret $sk_{TA}$ and public key $pk_{TA}$, TA executes CertS.Setup() and sets $\langle sk_{TA},CertS.params\rangle \leftarrow$ CLS.Setup(), then output CertS.params.
3. In order to keep a record of each vehicle $v_{i}$, TA set a pseudonym $alias_{v_{i}}$ to every $v_{i}$ based on its real identity $5GID_{v_{i}}$.
4. Each $RSC_{j}$, $DV_{j}$ and $DMV_{k}$ registers to TA, then computes CertS private keys as follows:
  - $RSC_{j}$, $DV_{j}$ and $DMV_{k}$ computes $Sec_{1,RSC_{j}}\leftarrow$ CertS.Secret($RSC_{j}$), $Sec_{1,DV_{j}}\leftarrow$ CertS.Secret($DV_{j}$) and $Sec_{1,DMV_{k}}\leftarrow$ CertS.Secret($DMV_{k}$), and makes a request for partial private key to the TA, respectively.
  - TA provides $Sec_{2,RSC_{j}}\leftarrow$ CertS.PartialK($sk_{TA}$, $RSC_{j}$, $P_{RSC_{j}}$); $Sec_{2,DV_{j}}\leftarrow$ CertS.PartialK($sk_{TA}$, $DV_{j}$, $P_{DV_{j}}$) and $Sec_{2,DMV_{k}}\leftarrow$ CertS.PartiaK($sk_{TA}$, $DMV_{k}$, $P_{DMV_{k}}$) to each entity securely.
  - $RSC_{j}$, $DV_{j}$ and $DMV_{k}$ set $\langle sk_{RSC_{j}},pk_{RSC_{j}}\rangle \leftarrow$ CertS.SKey($Sec_{1,RSC_{j}}$, $Sec_{2,RSC_{j}}$); $\langle sk_{DV_{j}},pk_{DV_{j}}\rangle \leftarrow$ CertS.SKey($Sec_{1,DV_{j}}$, $Sec_{2,DV_{j}}$) and $\langle sk_{DMV_{k}},pk_{DMV_{k}}\rangle \leftarrow$ CertS.SKey($Sec_{1,DMV_{k}}$, $Sec_{2,DMV_{k}}$), respectively.
5. Likewise, every $v_{i}$ computes $Sec_{1,v_{i}}\leftarrow$ CertS.Secret($alias_{i}$), TA provides $Sec_{2,v_{i}}\leftarrow$ CertS.PartialK($sk_{TA}$, $alias_{i}$, $P_{v_{i}}$), then $v_{i}$ sets $\langle sk_{v_{i}},pk_{v_{i}}\rangle \leftarrow$ CertS.SKey($Sec_{1,v_{i}}$, $Sec_{2,v_{i}}$).
6. $DMV_{k}$ selects a given universe of attributes U={1,…,N}, computes ABE parameters as $\langle ABE.amk,labe.params\rangle \leftarrow$ ABE.Setup(), then avails labe.params to the whole system. Note that DMVs will later send ABE.amk to TA in non busy hours.

### 5.2. Periodic Credential Generation

Periodically (on a daily basis), the vehicles request a road reporting credential (RReq) which permits the reporting of the abnormal scenes captured by the vehicle’s camera. To acquire a RReq from $DMV_{k}$, $v_{i}$ performs the following:

- $v_{i}$ composes a credential request message $RReq=\{alias_{i},K,ts\}$ where ts is the time stamp and *K* is a secret key to be used later.
- $v_{i}$ sends $C_{1}=Enc_{PK_{DMV_{k}}}\left\{RReq,pk_{v_{i}},\delta_{i}\right\}$ to the $DMV_{k}$, where δ is the signature for the RReq set as $\delta_{i}$ = CertS.Sign(RReq, $sk_{v_{i}}$).
- Upon receiving the message $C_{1}$, $DMV_{k}$ first decrypts $C_{1}$ using its private key, then verifies the signature as CertS.Verify(RReq, $alias_{i}$, $pk_{v_{i}}$, δ).

If it holds, $DMV_{k}$ generates reporting credential (*R*-cred) as follows:

1. Generate *R*-$cred=\langle alias_{i},exp,KN,\omega_{v_{i}}\rangle$ where exp is the expiration date, $\omega_{v_{i}}$ the set of attributes and KN the keyword for the credential. Note that KN is not specific for each credential but is the same based on the access structure.
2. $DMV_{k}$ sends $C_{1}=Enc_{K}(R$-cred) to $v_{i}$. Then, $v_{i}$ can recover *R*-cred by decrypting the $C_{1}$ under the shared secret key *K*.
3. $DMV_{k}$ sends periodically a list $List_{KN}$ of all the keywords enclosed in the credentials to RSCs.

### 5.3. On-Duty Token Generation

In the same way, $DMV_{j}$ generates on-duty token for the designated vehicles. These tokens authorize the DVs to recover the reported videos. For instance a police vehicle can get an *on-duty token* which extends its duty from police’s duties to basic ambulance’s duties. If an area *A* witnesses numerous accidents, several ambulances would go to the accident’s scenes in area *A* which might cause a temporal non-availability of ambulances. In that case, some of the police agents which have basic medical skills can attend to accident’s victims as they wait for the ambulances to arrive.

1. $DV_{i}$ composes an on-duty token request $DTRq=\{DV_{j},K_{1},ts\}$ where ts is the time stamp and $K_{1}$ is a secret key to be used later.
2. $DV_{i}$ sends $C_{2}=Enc_{PK_{DMV_{k}}}\left\{DTReq,pk_{v_{i}},\delta_{DV}\right\}$ to the $DMV_{k}$, where $\delta_{DV}$ is the signature for the DTRq set as $\delta_{DV}$ = CertS.Sign(DTRq, $sk_{DV}$).
3. Upon receiving the message $C_{2}$, $DMV_{k}$ first decrypts $C_{2}$ using its private key, then verifies the signature as CertS.Verify(DTRq, $DV_{j}$, $pk_{DV_{j}}$, $\delta_{DV}$).
4. $DMV_{j}$ set $\bar{D}\leftarrow$ ABE.KGN(ABE.amk, $AS_{DV_{j}}$) where $AS_{DV_{j}}$ is the access structure corresponding to the designated vehicle’s type (police or ambulance).
5. $DMV_{j}$ composes a token message $Tok_{DV}=\left\{\bar{D},exp,RK\right\}$ where exp is the expiring date, RK a shared secret which is given to DVs that are supposed to work within a defined geographic zone. Note that in real word, one police vehicle can be assign to attend to all the requests from a defined geographic area (three or five consecutive streets). $DMV_{j}$ send it to $DV_{j}$ encrypted under the shared symmetric key $K_{1}$ as $C_{3}=Enc_{K_{1}}\left(Tok_{DV}\right)$.

### 5.4. Abnormal Video Recording

We assume that the in-built camera has the functionalities which can allow the driver to upload a given file or the camera’s sensors can decide to upload a particular video after analyzing abnormal movements within the video [49]. The timing and circumstances techniques in which the video should be uploaded are not within the scope of this paper. The vehicles perform the following before uploading the video file captured by the camera:

1. After recording a video file, $v_{i}$ composes a message $V=\{Vfile_{j},KN\}$; uses the access policy $\omega_{v_{i}}$ retrieved in the credential *R*-cred and encrypts the file under the given attribute set $\omega_{v_{i}}$ as $SF_{j}\leftarrow$ ABE.Encrypt($Vfile_{j}$, $\omega_{j}$, labe.params).
2. $v_{i}$ sends $C_{4}=Enc_{PK_{RSC_{j}}}\left\{alias_{i},KN,SF_{j}\right\}$ to $RSC_{j}$.
3. $RSC_{j}$ decrypts $C_{4}$ and check if {$KN\in List_{KN}$}.
4. If not $C_{4}$ is discarded. Note that KN value are similar for all the vehicles which have a similar set of attribute such as ω. As mentioned before, $DMV_{j}$ can choose to give a type of attributes to a vehicle based on different criteria such accident record and reckless driving record. The choice of those criteria is beyond the scope of this paper.
5. Otherwise, $RSC_{j}$ forwards the file securely to neighboring RSCs.
6. $RSC_{j}$ generates $C_{5}^{{}^{\prime }}=\delta_{RSC_{j}}$ = CertS.Sign($SF_{j}$, $sk_{RSC_{j}}$) and broadcast $C_{5}=Enc_{RK}\left(C_{5}^{{}^{\prime }}\right)$ within its coverage area.

After receiving the beacons, DVs performs the following:

- $DV_{j}$ decrypts $C_{5}=Enc_{RK}\left(C_{5}^{{}^{\prime }}\right)$ using the area shared key of RK. Note that only DVs assigned to work within $RSC_{j}$ coverage can decrypt the $C_{5}$.
- $DV_{j}$ runs CertS.Verify($SF_{j}$, $RSC_{j}$, $pk_{RSC_{j}}$, $\delta_{RSC_{j}}$).
- $DV_{j}$ runs ABE.Decrypt($SF_{j}$, $\bar{D}$, labe.params) to get the original file of $Vfile_{j}$.

## 6. Performance Evaluation

In this section, we show the performance evaluation results of the proposed protocol based on security analysis, computational delay and communication overhead.

### 6.1. Security

The security achievements of the proposed protocol are as follows:

- *Authentication*: The authentication for every $v_{i}$ requesting a service file is provided by the certificateless signature scheme on message
  $RReq=\{alias_{i},K,ts\}$ with $C_{1}=Enc_{PK_{DMV_{k}}}\left\{RReq,pk_{v_{i}},\delta_{i}\right\}$ . No malicious user can falsify a valid signature based on the hardness of DL problem. Otherwise the verifier could check the validity of the message by running CLS.Verify(*m*, id, $pk_{id}$, σ) to check if $r\cdot \left(R+h_{2}\cdot P\right)\overset{?}{=}P_{id}+R_{id}+\left(h_{1}\;\cdot P_{pub}\right)$. Thus, the authentication is guaranteed for the proposed protocol.
- *Authorization*: Every vehicle has to get a periodic credential before it can participate in video reporting. $v_{i}$ sends $C_{1}=Enc_{PK_{DMV_{k}}}\left\{RReq,pk_{v_{i}},\delta_{i}\right\}$ to the $DMV_{k}$ to request a credential. After a valid verification, $DMV_{k}$ sends $C_{1}=Enc_{K}\left(R-cred\right)$ where $R-cred=\langle alias_{i},exp,KN,\omega_{v_{i}}\rangle$ as $v_{i}$’s credential which allows the $v_{i}$ to participate in video reporting.
- *Identity privacy preservation*: It is hard for an attacker to get a real identity of a vehicle within our proposed protocol. In the registration stage of the vehicle provided by TA, every vehicle $v_{i}$ is provided with a pseudo-identity $alias_{v_{i}}$. Though the malicious user would get the credential request message $RReq=\{alias_{i},K,ts\}$ , the single plain identity of $v_{i}$ which is available is its pseudo-identity $alias_{v_{i}}$. In the rest of the protocol, the remaining available information concerning $v_{i}$ is its pseudo-identity $alias_{v_{i}}$. We confirm that our protocol achieves identity privacy preservation.
- Fine-grained access control: In the proposed protocol, the video file Vfile sent to $v_{i}$ is encrypted under a set of attributes as $SF_{j}\leftarrow$ ABE.Encrypt($Vfile_{j}$, $\omega_{j}$, labe.params). The file is sent encrypted under the public key of $RSC_{j}$.$RSC_{j}$ only checks if $KN\in List_{KN}$; this will save the RSCs from availing bogus files to DVs. $RSC_{j}$ can not recover the file. Even for DVs, unless a $DV_{j}$ possesses the required secret shares $D_{leaf_{l}}=q_{leaf_{l}}\left(0\right)\cdot t_{i}^{-1}$ , it cannot reconstruct the root node *R* to be able to get the secret $q_{root}\left(0\right)\cdot k\cdot P=s\cdot k\cdot P$. Throughout the decryption stage based on the root or child node, except $DV_{j}$ has the obligatory secret shares, the decryption procedure returns ⊥. Consequently, even the entities (vehicles)that share a certain number of attributes can not conspire (collude) together to recuperate the secret that will achieves the decryption of the video file.
- Non-repudiation: A vehicle can not deny of participating in video reporting because the receiving $RSC_{j}$ keeps $v_{i}$’s pseudo identity and its anonymous keyword KN contained in the credential $R-cred=\langle alias_{i},exp,KN,\omega_{v_{i}}\rangle$.
- Traceability: Even though it is hard for an attacker to know the real identity of a vehicle, TA has the capability of revealing the vehicle’s real identity in case of disputes. TA makes a search to find which real identity corresponds to any given or reported $alias_{v_{i}}$. We conclude that the proposed protocol satisfies the traceability property.

### 6.2. Cost Comparison

We show the analysis results of the computational and communication costs of the proposed protocol.

#### 6.2.1. Computation Cost

When analyzing the computation cost of the proposed protocol, we deliberately omit the time complexity measurement of the setup phase since it is considered to be done offline and infrequently. We basically privilege the operations that dominate the speed of signature generation and verification. We adopt the implementation parameters in [50,51] with embedding degree 6, {G,q} represented by 161 bits and 160 bits respectively. The implementation was performed on a 3.5-GHz, core i-5, 16 GB RAM desktop computer with crypto++ library 5.6.5 [52]. The cost of respective security primitives are depicted in Table 2.

#### 6.2.2. Overall Cost Including Communication Cost

**Figure 3:**
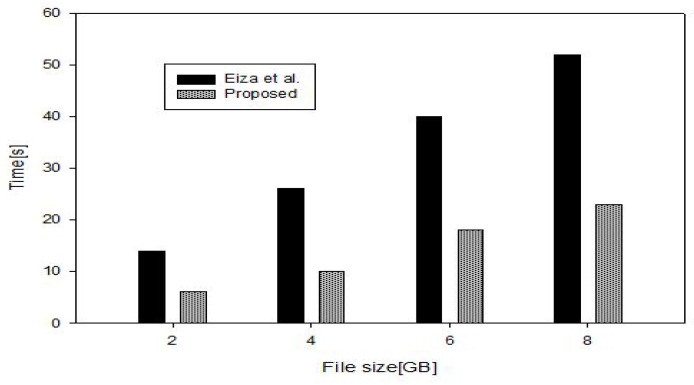
Enccryption/signing cost.

Note that TA uses secure symmetric encryption/decryption algorithm. For fairness in comparison, we adopt AES/CBC (256-bit key) with a processing speed of 65 MB/s. We also consider the size of the video ranging from 2 to 8 Gigabytes. We use SHA-512 hash function with a processing speed of 231 MB/s. The connection speed for the 5G-enabled vehicular network is set to 1.2 Gb/s and vehicle’s velocity to 100 km/h [53]. We also use CP-ABE toolkit [54] along with MIRACL [55] library to benchmark the performance of attribute-based encryption. We set the attribution number to 4 by following the reference [18]. The overall operation involved for signing and verifying is illustrated in Table 3. As being described in Section 5.4, $v_{i}$ computes $W_{i}=k\cdot P_{i}$ and $q_{leaf_{l}}\left(0\right)\cdot t_{i}^{-1}$ ; which equals to $\left(d+1\right)T_{mul}$ where *d* is the number of attributes based on the access structure. Furthermore, $v_{i}$ sends the file encrypted under the public of $RSC_{j}$ which equals to $T_{as-enc}$. $RSC_{j}$ only decrypts the file and check the validity of the KN which was earlier provided within the vehicle credential. In Figure 3, we show the time overhead for encrypting and signing the files recorded by the vehicles’ OBUs.

**Figure 4:**
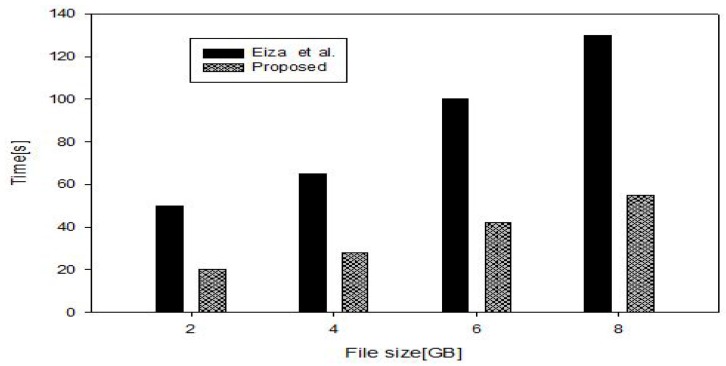
Overall cost.

**Figure 5:**
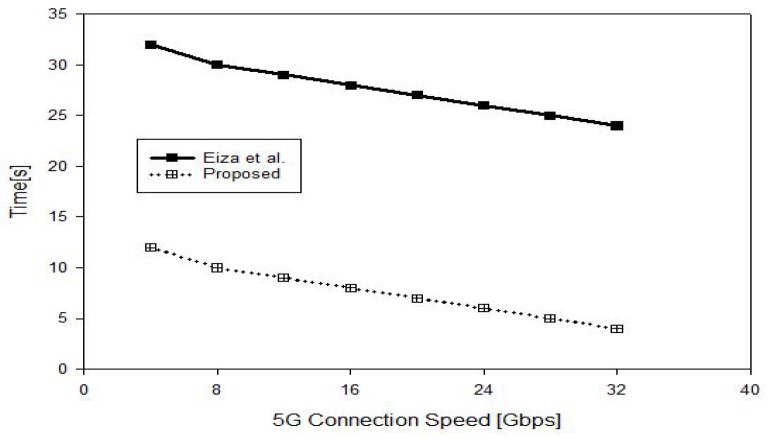
Overall cost under different 5G connection speeds.

To recover the video file, $DV_{i}$ decrypts the file in $T_{as-dec}$ and checks if $r\cdot \left(R+h_{2}\cdot P\right)\overset{?}{=}$$P_{id}+R_{id}+\left(h_{1}\;\cdot P_{pub}\right)$ for signature verification in $2T_{mul}$. $DV_{j}$ computes $q_{leaf_{l}}\left(0\right)\cdot k\cdot P$ in $dT_{mul}$. On the other hand, the protocol in [18] requires $T_{pair}+T_{mul}$ to perform *public key encryption with key search* [56]; $\left(d+1\right)T_{mul}+\left(d+1\right)T_{pair}$ for attribute-based encryption and $T_{mul}$ for signature generation. In order to recover the video file, the designated vehicle requires $3T_{pair}+4T_{mul}$ for certificate and signature verification and $4T_{pair}$ for attribute-based decryption. The total overhead which comprises the required time to encrypt, sign, transmit, verify and decrypt the reported file is shown in Figure 4. It is predicated that the connection speed for the 5G-enabled vehicles will be higher than 1.2 GB/s up to 1 Tb/s which has been already achieved for stationary wireless connection [57]. In Figure 5, we show the overall time overhead with different 5G connection speeds for a 2 GB video file. As shown in Figure 4, the time for the vehicle’s OBU to encrypt, sign and decrypt the recorded file in the proposed protocol is also less than the Eiza-Ni-Shi protocol [18] by 50%. These observations show that the proposed protocol offers the possibility to the vehicles that witnessed abnormal events such as road’s accidents to report the scene to the designated entities for a timely response.

## 7. Conclusions

In this paper, we noted that although there exist a few research works for secure video reporting service in 5G-enabled vehicular networks, their usage is limited because of public key certificates and expensive pairing operations are required. To overcome the limitation, we proposed a new secure and lightweight protocol for cloud-assisted video reporting service in 5G-enabled vehicular networks. Based on a fined-grained access control, the proposed protocol allowed the designated vehicles to recover the recorded video files without any prior communication. By providing security and privacy for the participating entities, the proposed protocol prevents malicious users from tracking, revealing or impersonating the system entities. Also, by using a new certificateless signature scheme, the proposed protocol assured the authentication of legitimate vehicles. With anonymous credentials instead of public key certificates, the proposed protocol guaranteed the authorization of participating entities. From the security and performance analysis results, we showed that the proposed protocol took a lightweight overhead compared to the state-of-the-art works. From these analysis results, we believe that the proposed protocol will help to realize timely and secure cloud-assisted video reporting service over 5G-enabled vehicular networks.

## Figures and Tables

**Table 1:** Terminology.

*Term*	Notation
$5G_{ID}$	Unique 5G identity for each OBU’s vehicle
TA	Trusted Authority
$DMV_{k}$	Department of Motor vehicle’s server
$RSC_{j}$	Roadside cloud’s server
$DV_{j}$	Identity of designated vehicle’s OBU
*R*-cred	$v_{i}$’s credential issued by $DMV_{k}$
KN	Key word within a vehicle’s credential
$List_{KN}$	List of generated KNs periodically
$Tok_{DV}$	A duty token for $DV_{j}$ generated by $DMV_{j}$
$alias_{i}$	$v_{i}$’s pseudo identity
ts	time stamp
$\delta_{i}$	certificateless signature of entity *i*
G	Elliptic curve group with the same order *q*
$P\in G_{1}$	A generator of $G_{1}$
$sk_{id},pk_{id}$	private, public key pair of an entity X
$t_{i}$	Master secret for each attribute *i*
ABE.amk	Attribute master key
$T_{i}$	Public key for each attribute i∈U
$alias_{v_{i}}$	$v_{i}$’s pseudonym
*U*	Universe of attribute
Γ	Access tree
ω	Attribute set
$AS_{j}$	Access Structure corresponding to entity *j*
*D*	Set of secret share $D_{leaf_{l}}$ in Γ
$Enc_{k}\left(.\right)$	Symmetric encryption under key *k*

**Table 2:** Measurement of cryptographic operations.

*Notation*	Operations	Time (ms)
$T_{pair}$	Bilinear pairing	4.5
$T_{mul}$	Point scalar multiplication	0.6
$T_{as-enc}$	Asymmetric encryption	1.17
$T_{as-dec}$	Asymmetric decryption	0.61
$T_{s-enc}$	Symmetric encryption	0.51
$T_{s-dec}$	Symmetric decryption	0.55
$T_{h}$	hash function	0.0001

**Table 3:** Computational costs of the Eiza-Ni-Shi protocol [18] and the proposed protocol.

Scheme Phase	Eiza et al. [18]	Proposed
Signing/video	$\left(d+2\right)T_{mul}+\left(d+2\right)T_{pair}$	$\left(d+1\right)T_{mul}$
Verification/video	$7T_{pair}+4T_{mul}$	$\left(d+3\right)T_{mul}$
Total cost/ms	64	7.2

(*d*=number of leaf node).

## References

[1] Shen X. (2015). Device-to-device communication in 5G cellular networks. IEEE Netw..

[2] Yu R., Ding J., Huang X., Zhou M.T., Gjessing S., Zhang Y. (2016). Optimal resource sharing in 5g-enabled vehicular networks: A matrix game approach. IEEE Trans. Veh. Technol..

[3] Andrews J.G., Buzzi S., Choi W., Hanly S.V., Lozano A., Soong A.C., Zhang J.C. (2014). What will 5G be?. IEEE J. Sel. Areas Commun..

[4] Li Q.C., Niu H., Papathanassiou A.T., Wu G. (2014). 5G network capacity: Key elements and technologies. IEEE Veh. Technol. Mag..

[5] Bhushan N., Li J., Malladi D., Gilmore R., Brenner D., Damnjanovic A., Sukhavasi R., Patel C., Geirhofer S. (2014). Network densification: The dominant theme for wireless evolution into 5G. IEEE Commun. Mag..

[6] Bloom C., Tan J., Ramjohn J., Bauer L. (2017). Self-driving cars and data collection: Privacy perceptions of networked autonomous vehicles. Proceedings of the Thirteenth Symposium on Usable Privacy and Security (SOUPS).

[7] Bellalta B., Belyaev E., Jonsson M., Vinel A. (2014). Performance evaluation of IEEE 802.11p-enabled vehicular video surveillance system. IEEE Commun. Lett..

[8] Vinel A. (2012). 3GPP LTE versus IEEE 802.11 p/WAVE: Which technology is able to support cooperative vehicular safety applications?. IEEE Wirel. Commun. Lett..

[9] Mir Z.H., Filali F. (2014). LTE and IEEE 802.11 p for vehicular networking: A performance evaluation. EURASIP J. Wirel. Commun. Netw..

[10] Chen S., Zhao J. (2014). The requirements, challenges, and technologies for 5G of terrestrial mobile telecommunication. IEEE Commun. Mag..

[11] Belyaev E., Vinel A., Surak A., Gabbouj M., Jonsson M., Egiazarian K. (2015). Robust vehicle-to-infrastructure video transmission for road surveillance applications. IEEE Trans. Veh. Technol..

[12] Eiza M.H., Ni Q., Owens T., Min G. (2013). Investigation of routing reliability of vehicular ad hoc networks. EURASIP J. Wirel. Commun. Netw..

[13] Eiza M.H., Owens T., Ni Q., Shi Q. (2015). Situation-aware QoS routing algorithm for vehicular ad hoc networks. IEEE Trans. Veh. Technol..

[14] Wang C.X., Haider F., Gao X., You X.H., Yang Y., Yuan D., Aggoune H., Haas H., Fletcher S., Hepsaydir E. (2014). Cellular architecture and key technologies for 5G wireless communication networks. IEEE Commun. Mag..

[15] Gai K., Qiu M., Tao L., Zhu Y. (2016). Intrusion detection techniques for mobile cloud computing in heterogeneous 5G. Secur. Commun. Netw..

[16] Chen S., Qin F., Hu B., Li X., Chen Z. (2016). User-centric ultra-dense networks for 5G: Challenges, methodologies, and directions. IEEE Wirel. Commun..

[17] Chatterjee S., Kar A.K., Gupta M. (2017). Critical Success Factors to Establish 5G Network in Smart Cities: Inputs for Security and Privacy. J. Glob. Inf. Manag..

[18] Eiza M.H., Ni Q., Shi Q. (2016). Secure and Privacy-Aware Cloud-Assisted Video Reporting Service in 5G-Enabled Vehicular Networks. IEEE Trans. Veh. Technol..

[19] Yoo S.G. (2017). 5G-VRSec: Secure Video Reporting Service in 5G Enabled Vehicular Networks. Wirel. Commun. Mob. Comput..

[20] Malhi A.K., Batra S. (2015). An efficient certificateless aggregate signature scheme for vehicular ad-hoc networks. Discrete Math. Theor. Comput. Sci..

[21] Horng S.J., Tzeng S.F., Huang P.H., Wang X., Li T., Khan M.K. (2015). An efficient certificateless aggregate signature with conditional privacy-preserving for vehicular sensor networks. Inf. Sci..

[22] Cho W., Park Y., Sur C., Rhee K.H. (2013). An Improved Privacy-Preserving Navigation Protocol in VANETs. JoWUA.

[23] Altayeb M., Mahgoub I. (2013). A survey of vehicular ad hoc networks routing protocols. Int. J. Innov. Appl. Stud..

[24] Yin J., ElBatt T., Yeung G., Ryu B., Habermas S., Krishnan H., Talty T. (2004). Performance evaluation of safety applications over DSRC vehicular ad hoc networks. Proceedings of the 1st ACM International Workshop on Vehicular Ad Hoc Networks.

[25] Hussain R., Son J., Eun H., Kim S., Oh H. Rethinking vehicular communications: Merging VANET with cloud computing. Proceedings of the 2012 IEEE 4th International Conference on Cloud Computing Technology and Science (CloudCom).

[26] Olariu S., Khalil I., Abuelela M. (2011). Taking VANET to the clouds. Int. J. Pervasive Comput. Commun..

[27] Hussain R., Abbas F., Son J., Oh H. TIaaS: Secure cloud-assisted traffic information dissemination in vehicular ad hoc networks. Proceedings of the 2013 13th IEEE/ACM International Symposium on Cluster, Cloud and Grid Computing (CCGrid).

[28] He W., Yan G., Xu L.D. (2014). Developing vehicular data cloud services in the IoT environment. IEEE Trans. Ind. Inform..

[29] Lee E., Lee E.K., Gerla M., Oh S.Y. (2014). Vehicular cloud networking: architecture and design principles. IEEE Commun. Mag..

[30] Nkenyereye L., Rhee K.H. (2015). Secure Traffic Data Transmission Protocol for Vehicular Cloud. Advances in Computer Science and Ubiquitous Computing.

[31] Nkenyereye L., Tama B.A., Park Y., Rhee K.H. (2015). A Fine-Grained Privacy Preserving Protocol over Attribute Based Access Control for VANETs. JoWUA.

[32] Nkenyereye L., Rhee K.H. (2015). Secure Taxi Service Scheme in Vehicular Cloud Environment. Int. Inf. Inst. (Tokyo) Inf..

[33] Zhang N., Cheng N., Gamage A.T., Zhang K., Mark J.W., Shen X. (2015). Cloud assisted HetNets toward 5G wireless networks. IEEE Commun. Mag..

[34] Trivisonno R., Guerzoni R., Vaishnavi I., Soldani D. (2015). SDN-based 5G mobile networks: Architecture, functions, procedures and backward compatibility. Trans. Emerg. Telecommun. Technol..

[35] Mantas G., Komninos N., Rodriuez J., Logota E., Marques H. (2015). Security for 5G communications. Fundamentals of 5G Mobile Networks.

[36] Yang N., Wang L., Geraci G., Elkashlan M., Yuan J., Di Renzo M. (2015). Safeguarding 5G wireless communication networks using physical layer security. IEEE Commun. Mag..

[37] Alam M., Yang D., Rodriguez J., Abd-alhameed R. (2014). Secure device-to-device communication in LTE-A. IEEE Commun. Mag..

[38] Lin X., Sun X., Ho P.H., Shen X. (2007). GSIS: A secure and privacy-preserving protocol for vehicular communications. IEEE Trans. Veh. Technol..

[39] Raya M., Papadimitratos P., Aad I., Jungels D., Hubaux J.P. (2007). Eviction of misbehaving and faulty nodes in vehicular networks. IEEE J. Sel. Areas Commun..

[40] Dikaiakos M.D., Florides A., Nadeem T., Iftode L. (2007). Location-aware services over vehicular ad-hoc networks using car-to-car communication. IEEE J. Sel. Areas Commun..

[41] Sampigethaya K., Li M., Huang L., Poovendran R. (2007). AMOEBA: Robust location privacy scheme for VANET. IEEE J. Sel. Areas Commun..

[42] Chim T.W., Yiu S., Hui L.C., Li V.O. (2014). VSPN: VANET-based secure and privacy-preserving navigation. IEEE Trans. Comput..

[43] Coronado E., Cherkaoui S. Provisioning of on-demand services in vehicular networks. Proceedings of the Global Telecommunications Conference (GLOBECOM).

[44] Li C.T., Hwang M.S., Chu Y.P. (2008). A secure and efficient communication scheme with authenticated key establishment and privacy preserving for vehicular ad hoc networks. Comput. Commun..

[45] Zhu H., Lu R., Shen X., Lin X. (2009). Security in service-oriented vehicular networks. IEEE Wirel. Commun..

[46] Yao X., Chen Z., Tian Y. (2015). A lightweight attribute-based encryption scheme for the Internet of Things. Future Gener. Comput. Syst..

[47] He D., Chen J., Zhang R. (2012). An efficient and provably-secure certificateless signature scheme without bilinear pairings. Int. J. Commun. Syst..

[48] Chin W.H., Fan Z., Haines R. (2014). Emerging technologies and research challenges for 5G wireless networks. IEEE Wirel. Commun..

[49] Birem M., Berry F. (2014). Dreamcam: A modular fpga-based smart camera architecture. J. Syst. Archit..

[50] Lu R., Lin X., Zhu H., Ho P.H., Shen X. (2009). A novel anonymous mutual authentication protocol with provable link-layer location privacy. IEEE Trans. Veh. Technol..

[51] Miyaji A., Nakabayashi M., Takano S. (2001). New explicit conditions of elliptic curve traces for FR-reduction. IEICE Trans. Fundam. Electron. Commun. Comput. Sci..

[52] Wei D. (2017). Crypto++ Library 5.6.5, a Free C++ Class Library of Cryptographic schemes. http://www.cryptopp.com.

[53] Demestichas P., Georgakopoulos A., Karvounas D., Tsagkaris K., Stavroulaki V., Lu J., Xiong C., Yao J. (2013). 5G on the horizon: Key challenges for the radio-access network. IEEE Veh. Technol. Mag..

[54] Bethencourt J., Sahai A., Waters B. Advanced Crypto Software Collection—Ciphertext-Policy Attribute-Based Encryption. http://acsc.cs.utexas.edu/cpabe/.

[55] MIRACL Crypto SDK, CertiVox UK, R.U. (2016). Multiprecision Integer and Rational Arithmetic Cryptographic Library. https://www.certivox.com/miracl.

[56] Baek J., Safavi-Naini R., Susilo W. Public key encryption with keyword search revisited. Proceedings of the International Conference on Computational Science and Its Applications.

[57] BBCNews (2015). 5G Researchers Manage Record Connection Speed. http://www.bbc.co.uk/news/technology-31622297.

